# Reversible Underwater Adhesion: The Unique C-shaped Suckers of Net-winged Midge Larvae (*Blepharicera* sp.)

**DOI:** 10.1038/s41598-020-66268-3

**Published:** 2020-06-10

**Authors:** Guan-Lin Liu, Haw-Kai Chang, Yung-Chieh Chuang, Yu-Min Lin, Po-Yu Chen

**Affiliations:** Department of Materials Science and Engineering, National Tsing Hua University, Hsinchu, 30013 Taiwan

**Keywords:** Bioinspired materials, Biomechanics

## Abstract

Aquatic insects living in fast-flowing streams have developed various types of attachment systems to resist being carried away by strong currents. Combinations of various attachment devices offer aquatic insects advantages in underwater adhesion on substrates with different surface properties. In this study, the net-winged midge (*Blepharicera* sp.) larvae were investigated to understand micro-/nano-structural attachment mechanisms. The hierarchical structure of insect adhesive surfaces was characterized using Optical Microscopy (OM), Scanning Electron Microscopy (SEM) and Transmission Electron Microscopy (TEM). Centrifugal measurements were also conducted to measure the critical rotational velocity at which the larvae of *Blepharicera* sp. can adhere to substrates with varying roughness. Commercial suckers require smooth substrate surface to maintain a pressure that is lower than the surrounding pressure for adhesion under the sucker cup while the suckers of net-winged midge larvae possess hierarchical micro-/nano-structures, which attach closely to rough surfaces underwater. Furthermore, the functions of microstructures observed on the sucker, including wrinkled surface, inward setae, outer fibers, and nick were explored and may contribute to underwater adhesion. The aligned C-shaped suckers can attach and detach effectively by closing or opening the gap. The unique microstructure and adhesion capability of such suckers could shed light on the design and synthesis of novel bio-inspired devices for reversible underwater adhesion.

## Introduction

Immature insects living in fast-flowing streams have to cope with the risk of being swept away and have evolved different attachment mechanisms. Living underwater can also help them escape from predators^1^. Petra Ditsche-Kuru^2^ classified the attachment mechanisms in the aquatic environment into four types: (i) suction, (ii) friction, (iii) secretions and glue, and (iv) mechanical principles such as hook, lock, clamp, and spacer. A sucker is a device that uses the difference between atmospheric pressure and inner pressure to maintain adhesion. Important properties of a sucker cup include flexibility, smooth and soft surface of its margin, and muscles or muscular fibers for generating a reduced pressure under the cup. Sucker devices have been documented from many soft-bodied animals^3^. However, only a few examples have been found among the Arthropoda.

Blephariceridae (or net-winged midges) larvae from the nematocerous family are known for their sucker attachment. They live in fast-flowing streams, attaching to stones by using six suction discs located on the ventral side of the body^4–7^. In order to aid suction, the attachment is supplemented by a secretion of the sternal glands. Many well-known marine animals utilize suction to attach to a suitable surface, such as limpets and abalones^8^, squid and octopuses^9,10^, and river loaches^11^. However, the larva of net-winged midges have not been fully investigated. Gregory W. Courtney^7^ conducted systematic and comparative studies on the natural history, morphological descriptions, and diagnoses of the genus *Blepharicera* Macquar net-winged midges from Eastern North America at the larvae, pupae, and adult stages. *Blepharicera* larvae show relatively little morphological variation among different species, which may due to the common need for adaptions to torrential habitats, such as the fused head, thorax, and first abdominal segment named cephalothorax, six ventral suctorial discs and specialized mouthparts. Kang *et al*.^12^ identified micro-structural features and proposed functional adaptations of suction attachment organs in two species of net-winged midge larvae (*L. cinerascens* and *L. cordata*). Each suctorial disc is covered with spine-like microtrichia and flat rim microtrichia, which may contribute to the seal on a variety of surfaces. The V-notch on the suction disc actively opened by muscles may provide quick detachment mechanism. Frutiger^13^ investigated locomotory behavior of three larval blepharicerids (*L. cinerascens cinerascens, L. cinerascens minor*, and *H. lugubris*) by high-speed video-macroscopy and digital image analyses. Six basic locomotion patterns were proposed: straight forward, backward, slow sideways, rapid sideways movements, re-orientation, and foraging. However, the net-winged midge larvae adhesion capability on varying surface roughness and the capillary force contribution have not been studied. In this study, we investigated the relationship between micro-/nanostructures and attachment mechanisms of the *Blepharicera* sp. larva. The suction disc hierarchical structure was characterized with optical microscopy, SEM, and TEM. Critical rotational velocities measured from centrifugal tests provided quantified adhesion capability data of *Blepharicera* sp. larvae on substrates with varying surface roughness and wettability. We hope this study can provide further understanding in biological underwater adhesion and offer inspirations for novel bio-mimetic underwater attachment devices and systems.

## Results and Discussion

### Macroscopic Observation of *Blepharicera* sp. Larvae

**Figure 1:**
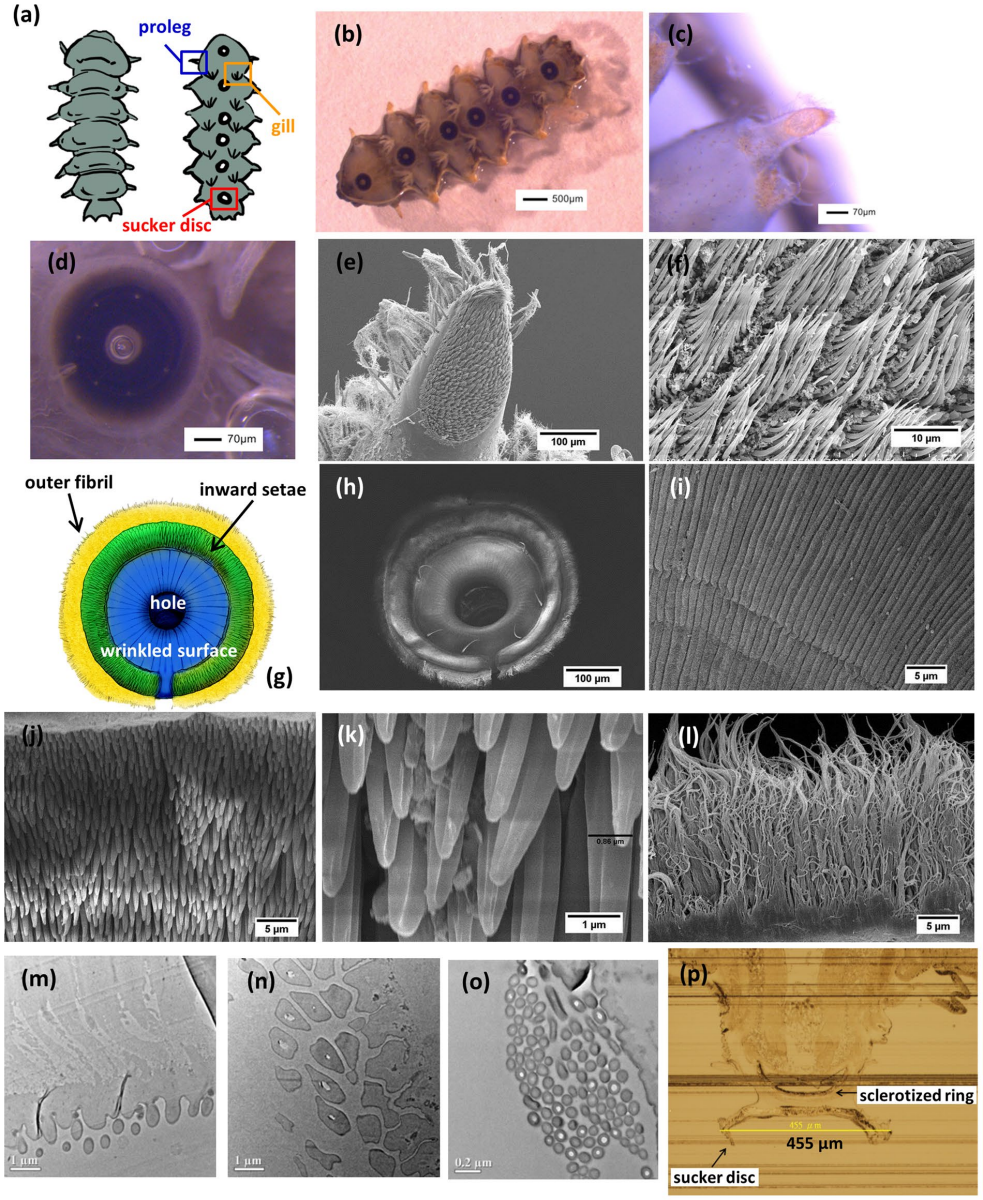
(**a**) Illustration of larva of *Blepharicera* sp. (Diptera) indicating that prolegs, gills and sucker discs can be observed in ventral side. (**b**) A larva of *Blepharicera* sp. attach under a glass slide. Morphological views of suckers. (**c**) Stereoscopic image of proleg of the larva of *Blepharicera* sp. and the sucker (**d**) attaching to a surface (47 μm in radius of the hole and 195 μm in radius of whole sucker) and (**e**,**f**) SEM images of proleg showing that setae are arranged in an array. (**g**) Illustration picture and (**h**) SEM image of a sucker of larva of *Blepharicera* sp., which can be divided into four parts: (**i**) wrinkled surface, (**j**,**k**) inward setae and **(l)** outer fibers. TEM images of (**m**) the layer of wrinkled surface revealing the thickness of which is ~1.5 μm, (**n**) the cross-section of the inward setae showing and hollowed structure and (**o**) the cross-section of the outer fibers indicating the diameter of which is ~0.05 μm. (**p**) The histological microtome image showing sucker disc and sclerotized ring.

Larvae of *Blepharicera* sp. live in the high flow rate rivers where oxygen concentration is high. They bear gills in the abdomen to breathe air in the water (Fig. 1a,b). There are six sucker discs in the first six segments of ventrally flattened bodies^7^. These six sucker discs are used to adhere to stones or riverbeds in rapid water flow and are utilized for locomotion. The first six segments also possess prolegs on two lateral sides^7^. Movements of the larva of *Blepharicera* sp., including forward, backward, and side motions, are observed in this study. Supplementary Video S1 shows that the larva of *Blepharicera* can move forward with one of the suckers detaching and the other five suckers attaching to the surface. This movement seems to assure that the *Blepharicera* sp. larva firm adhesion on the surface while it is still able to move forward simultaneously. The average number of attached suckers during the forward movement is 2.68 and it is observed that the larvae kept at least two suckers attached during all types of locomotion to ensure safe attachment and allow maximal mobility simultaneously^13^. The larva of *Blepharicera* sp. is capable of moving backward and sideways (Supplementary Video S2). However, it seems that the backward movement is not as fluent and efficient as the forward movement of the *Blepharicera* sp., while Frutiger^13^ observed that the *L. cinerascens cinerascens* could move faster backward compared to the other movements. In addition, the rapid side motion by alternatively moving anterior and posterior suckers is also observed when the *Blepharicera* sp. larva encounters stimulation, such as rapid flow (Supplementary Video S3).

### Microscopic Observation and of Larva of *Blepharicera* sp

An individual sucker disc and a proleg were observed under OM, SEM, and TEM. The microstructures of the sucker disc and the proleg are shown in Fig. 1c–p. The sucker disc (Fig. 1d) of the *Blepharicera* sp. larva can be classified into four parts from inside out: central hole, wrinkled surface, inward setae, and outer fibers. Different colors are used to label these four segments (Fig. 1g). The hole located at the center of the sucker disc (Fig. 1g) and the diameter is ~100 μm (Fig. 1h). The outside of the hole is the wrinkled surface, which has many stripes aligned radially from the periphery of the hole. The width of each stripe is ~1 μm (Fig. 1i) and the thickness of wrinkled surface is ~1.5 μm (Fig. 1m). Connected to the wrinkled surface are the inward setae, all pointing toward the center of the sucker disc and are ~1 μm in radius (Fig. 1j,k). Figure 1n shows the hollowed structure of the inward setae. The outmost part is the outer fibers (Fig. 1l) and is ~0.05 μm in diameter (Fig. 1o). Unlike the inward setae, the outer fibers point outward. Compared to the microstructural feature observed on the suckers of the other two net-winged midge larvae (*L. cinerascens* and *L. cordata*)^12^, the inward setae (Fig. 1j,k) and outer fibers (Fig. 1l) of the *Blepharicera* sp. show similar morphology and dimensions. The flattened microtrichia rim found in the *L. cordata*^12^ is not observed in the *Blepharicera* sp. The radial wrinkle patterns (Fig. 1i) seem to be distinct from the sucker discs of the two blepharicerid larvae investigated by Kang *et al*.^12^

Figure 1c shows the microstructure of the *Blepharicera* sp. larva proleg under stereoscopy observation. SEM images (Fig. 1e,f**)** further indicate that the surface of proleg consists of many setae arranged in an array. These setae may have a function as frictional devices, which can supplement the adhesion mechanism of the *Blepharicera* sp. larva. Each seta is ~1 μm in diameter, which is similar size to the seta on the sucker.

### Influence of the Surface Roughness and Wettability on Suction

**Figure 2:**
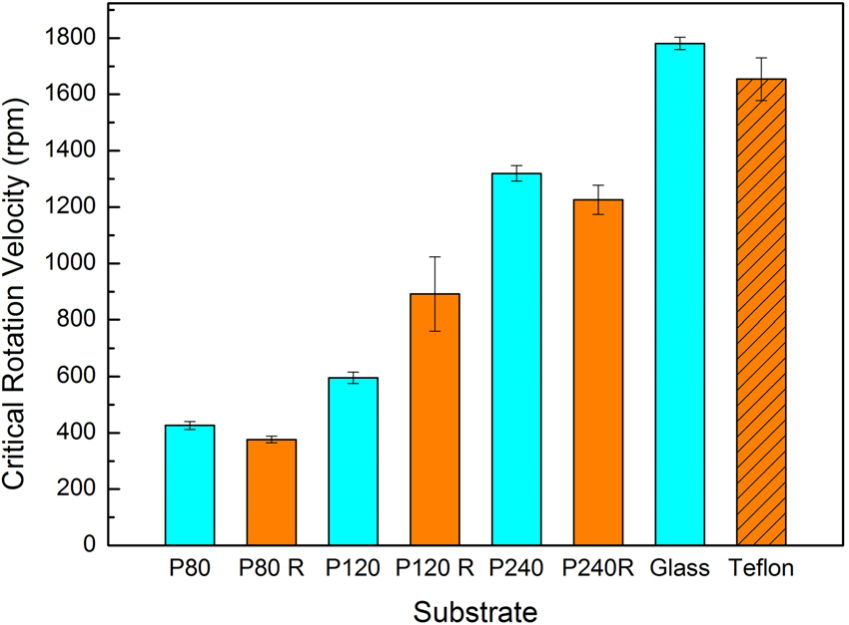
Critical rotational velocities versus substrates with varying surface roughness and wettability. Results show that the suckers perform better adhesion ability with decreasing surface roughness. The blue bars are the PDMS replicas of original mold sand paper (red bars on the left). The sand paper is more hydrophilic than its replicas. The glass slide is hydrophilic whereas the Teflon is hydrophobic, both of which are smooth (with roughness <5 μm.).

Figure 2 shows the critical rotational velocities that the *Blepharicera* sp. larva could resist on different substrates. It is well-known that suckers work better on smooth surfaces^3,14,15^. The centrifugal measurements reveal that the sucker has the best adhesion performance on the glass slide and the Teflon, which have the smoothest surfaces among the substrates tested (the critical rotational velocities are higher than 1600 rpm). On the roughest surfaces, such as P80 sandpaper and its PDMS replica, the sucker shows poor adhesion capability, reducing to ~400 rpm. The result indicates that the adhesion mechanism of a sucker is closely related to the roughness of a surface, but does not depend on the surface wettability. It is known that attachment devices utilizing capillarity interaction typically have better adhesion ability on hydrophilic surfaces than on hydrophobic ones, as shown by Huber *et al*.^16^ and Lin *et al*.^8^. For suckers, capillarity does not seem to contribute greatly to the adhesion. The *Blepharicera* sp. larva shows significantly weaker adhesion capability with increasing surface roughness. It is apparent that the main adhesion mechanism of the sucker is by suction via the reduced inner pressure. In dry adhesion cases, only mechanical interlocking, elastic forces, chemical bonding and van der Waals forces can work, while in wet adhesion cases capillary forces and viscous forces contribute considerably to adhesion^2^. Capillary forces work when fluid film is involved. Fully immersed aquatic environment, capillary forces would not play an important role in adhesion. Our experiment condition is in air with wet contacting surfaces. We found that wettability, or surface tension of the substrates, may have minor influence on the underwater adhesion of the larva of *Blepharicera* sp.

Our results from the centrifugal experiments suggest that the roughness, not the wettability of surface, dominates the adhesion capability. We did not directly measure “adhesion force”, instead, the critical rotational velocity at which insects can no longer attach to the surface is measured. Adhesion is more related to friction. If the solid has a high friction coefficient, it usually has a high adhesion property^17^.

### Constructional Properties of Sucker

**Figure 3:**
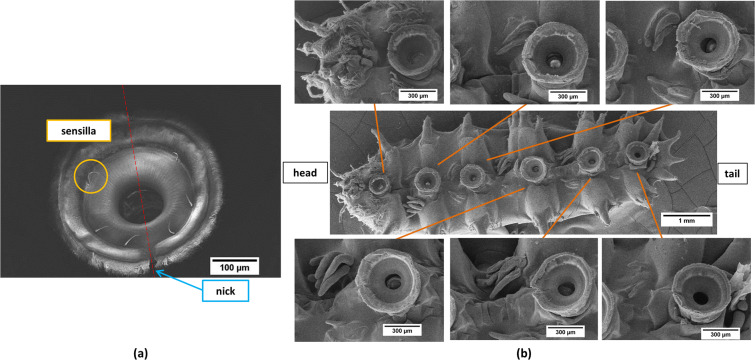
(**a**) Six sensilla are on the rim of the wrinkled surface part of the larva of *Blepharicera*’s sucker. The sensilla locate symmetrically along the central line through the nick. (**b**) SEM images of the larva of *Blepharicera* sp. show that the nicks are aligned in a direction toward the head.

Sensory equipment on attachment devices has important functions. Insects use sensory equipment to monitor the surface and external forces in the contact area. These sensory organs include sensilla, which are most likely strongly modified arthropod setae. For the mayfly *Epeorus assimilis* larvae^18^, sensilla can be found on the front part of the strong and curved claws. These sensilla are assumed to help detect irregularities on the surface of the substrates. Six sensilla can be observed on the rim of the wrinkled surface part of the sucker in the *Blepharicera* sp. larva. Each sucker has a nick and the sensilla locate symmetrically with the line through the nick (Fig. 3a). Those nicks are aligned in a direction toward the head (Fig. 3b). The histological microtome image shows that the sucker disc is like a shallow container, which might help increase contact area (Fig. 1p). In Gregory’s findings^7^, the shape and distribution of dorsal sensilla is highly consistent among different species of Blepharicera despite their diverse habitats. Future research should investigate how sensilla on the sucker differ among species. On the base of the sucker disc is a cup-shaped sclerotized ring, providing the strong structure to bear the low pressures when the suckers develop attachment^5^.

**Figure 4:**
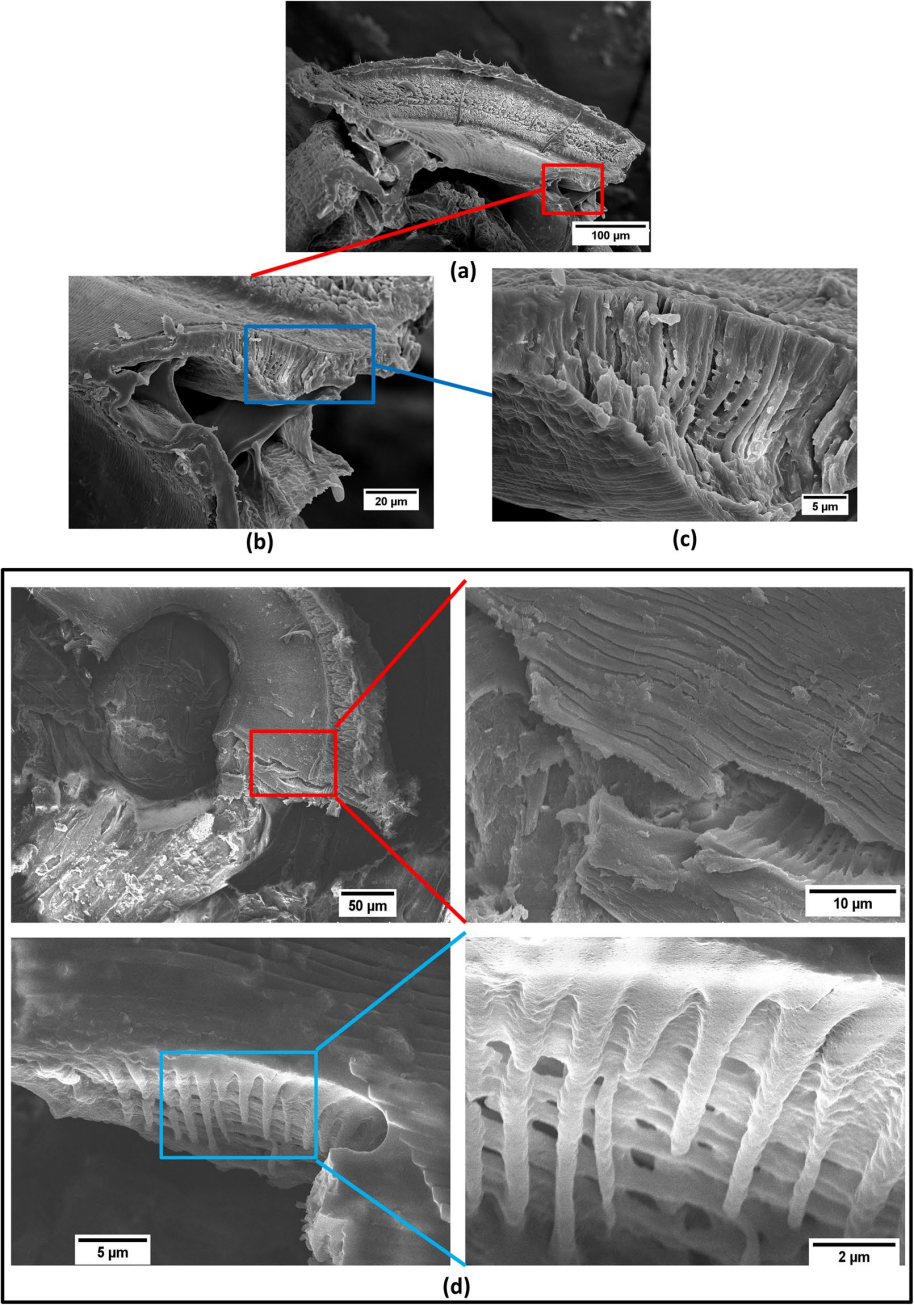
(**a**) The inner architecture of the sucker is similar to donut shape, which is hollowed inside. This architecture might be the tunnel for fluid to flow through, or provide flexibility for the sucker and helps it to adapt to a variety of surface profiles. (**b**,**c**) The alignment of fibers is perpendicular to the top wrinkled surface. (**d**) SEM micrographs at higher magnifications showing interwoven structure of the fibers can be observed. This interwoven structure might provide the strength of the sucker.

The inner architecture of the sucker is donut shaped and hollow and might form a tunnel for fluid to flow through (Fig. 4a). The tunnel is ~30 μm in diameter and the cross-section is triangular. In the adhesive pads of insects, a porous inner structure is common and is believed to provide flexibility for the adhering device and help it to adhere or attach to a variety of surface profiles^19^. The inner tunnel structure of the *Blepharicera* larva’s sucker might have the same function. When making contact to the surface, the adhesive pads can change their shape under loading^20^. Furthermore, alignment of fibers underneath is perpendicular to the top surface of the sucker tunnel (Fig. 4b,c). Under higher magnification, we can observe that the alignment of fibers is actually an interwoven structure (Fig. 4d). These fibers are ~500 nm in diameter, together composing the adhesive pads of the sucker. Spaces and pores can be observed among the fibers. The interwoven plywood structure of fibers may lead to the enhancement in mechanical property of the adhesive pads.

In order to investigate the gait of the *Blepharicera* sp. larva, we dropped red ink on the larva while it was creeping on the surface of the glass slide. After two hours, red ink had dried and we observed the footprint morphology under the OM. The OM image of one footprint shows that there is a white ring, which has fewer red ink around the sucker, revealing that red ink could not flow across this region (Supplementary Fig. S1). This might be due to the tight contact between the inward setae and the surface. However, the central part of the sucker shows red, indicating that red ink can flow into the sucker.

### Attachment Behavior

Red ink was also selected as a staining fluid. Fluid enters the sucker through the nick when the sucker is about to detach (Supplementary Video S4), demonstrating that the nick is the entrance or exit of the sucker. When the sucker firmly attaches to the surface, fluid flows around the periphery of the sucker and cannot penetrate into the sucker. The outer fibers probably prevent the invasion of fluid (Supplementary Video S5), which assures the reduced inner pressure of the sucker.

In summary, the microstructures all contribute to the suction function. There are three possibilities explaining the wrinkled surface of the sucker. One is that the wrinkled surface provides flexibility, or increases the friction coefficient, both of which could improve performance under submerged conditions. The second is that the wrinkled surface serves as the channels between the sucker and substrate for water leakage. The last one suggests a similar function as denticles and radial grooves on the octopus tentacle sucker^9,21^, which provide a waterfilled network of spaces that transmit the subambient pressure in the cavity of the sucker to the outside, leading to reduced inner pressure.

**Figure 5:**
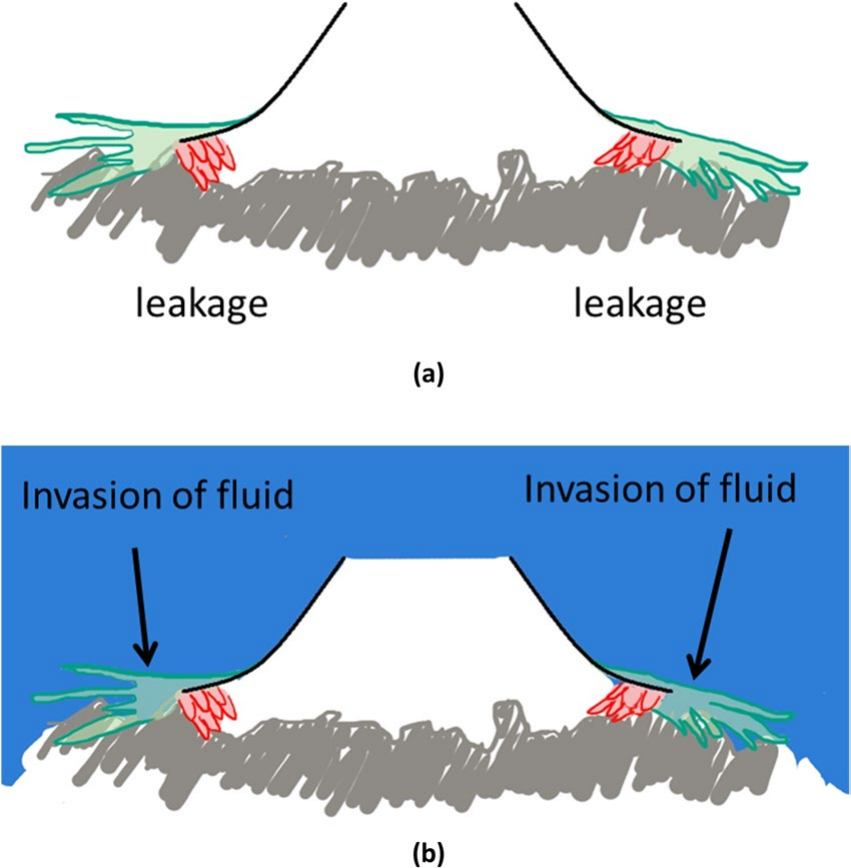
Illustrations showing the functions of inward setae (red) and outer fibers (green). (**a**) Inward setae can help attach to the rough surfaces and compensate the leakages. (**b**) Outer fiber can prevent the invasion of fluid.

Due to the irregularities on the surface, it is difficult for a normal sucker to attach to a rough surface compared with a smooth one. The inward setae can interlock with the rough surface and help seal the sucker (Fig. 5a). Furthermore, one of the obstacles of underwater adhesion is that water would flow into the adhering device and thus prevent adhesion. The outer fibers have proved to prevent water flow from invasion (Fig. 5b).

## Conclusions

The major discoveries of this study are summarized as follows:

1. The sucker of the larva of *Blepharicera* sp. can be divided in to four parts: central hole, wrinkled surface, inward setae, and outer fibers. Each sucker has a nick, serving as the entrance or exit for waterflow. The function of the wrinkled surface might be: (1) providing flexibility or increasing friction coefficient, both of which could improve the performance of attaching under submerged conditions, (2) serving as the channels between the sucker and the substrate for water leakages, and (3) providing a waterfilled network of spaces that transmit the sub-ambient pressure in the cavity of the sucker to the outside, leading to reduced inner pressure. The inward setae fill the cavities on rough surfaces, which might lead to a tighter contact between the sucker and the substrate. Furthermore, the outer fibers can prevent fluid from invasion, resulting in the maintenance of reduced inner pressure.

2. The sucker of the *Blepharicera* sp. larva is composed of microconstituents (wrinkled surface, inward setae, and outer fibers) on it. The inner architecture of the sucker has a donut shape which is hollowed inside. This donut shape might function as a tunnel for fluid to flow through or might provide flexibility for the sucker and assist the sucker in adapting to a variety of surface profiles.

3. The sucker shows stronger adhesion capability with decreasing surface roughness. However, there is no significant difference between the adhesions onto either hydrophilic or hydrophobic surfaces, indicating that capillarity does not contribute to underwater adhesion of the sucker. Instead, surface roughness has significant influence on the adhesion of the sucker of the *Blepharicera* sp. larva.

## Methods

### Materials

Larvae of *Blepharicera* sp. were collected from streams in Neiwan District (Hsinchu, Taiwan) and were transported alive to the laboratory for investigation. Larvae were kept in fresh water and the transportation time was minimized to approximately 1 hour.

### Macroscopic Observation

Larvae were kept in an 11.5 cm × 11.5 cm × 20 cm water tank after captured. An air pump (Hidoli HID-J-101, Fu Siang Pet Co., Taichung, Taiwan) was set inside the water tank to pump air into water. The movement and locomotion of aquatic insects were recorded with a digital camera (Panasonic LUMIX DMC-FX50).

### Optical Microscopy and Stereoscopy

Larvae immersed in 70% ethanol were examined under stereoscope and optical microscope to observe their microstructures in ventral and dorsal view. Stereoscopic images were taken with Olympus SZX7 Zoom Stereomicroscope (Olympus Co., Tokyo, Japan) equipped with a CCD camera (Infinity 1, Lumenera Co., Ontario, Canada). The magnification of stereoscope ranged from 8X to 56×. The movement and attaching behavior of aquatic insects were also recorded with CCD camera under stereoscope.

In order to observe the function and mechanism of sucker underwater, we used red ink to make fluid noticeable and recorded the video with the CCD camera under stereoscope. Additionally, we utilized red ink to mark the footprints of the larva of *Blepharicera* sp. to elucidate the microstructural architecture and function of the sucker.

### Scanning Electron Microscopy

Specimens for SEM (Scanning Electron Microscope) observation were prepared with the following procedures. The collected larvae were dehydrated using a CPD (Critical Point Dryer). Critical point drying^22^ is a method to dehydrate the specimen without shrinkage or change in shape. To prevent deformation of biological samples during the drying process, a fixation process is required before drying by immersing the collected larvae in Karnovsky’s fixative^23,24^ (the main component is paraformaldehyde). The samples were then rinsed in 0.2 M cacodylate buffer to wash out residual fixative. After fixation, the samples were immersed in ethanol solutions with different concentrations, from 30% to 100%. The samples were then positioned in the chamber of CPD (Tousimis Samdri-795, MD, USA) for the final dehydration.

Microstructural characterization of the attachment devices was observed by a Cold-Emission FE-SEM (Hitachi SU-8010). The CPD treated specimens were sputter coated with a thin layer of platinum to enhance electron conductivity on the surface. Secondary electron images (SEI) were taken at an accelerating voltage of 10 kV and a working distance of 10 mm.

### Histological Microtome and Transmission Electron Microscopy

Samples were submerged in pre-fixation (2% Paraformaldehyde, 2.5% Glutaraldehyde) overnight, and post-fixed in 1% OsO4 in distilled water for 1 h. Then the samples were dehydrated with different ethanol and acetone concentration. After dehydration, the sample was mounted into an epoxy resin and cut extremely thin cross-section slices of suckers by microtome. Finally, lead citrate was used to improve the image contrast for further Transmission Electron Microscopy (JEOL JEM-2100F) observation.

### Adhesion Capability Evaluation

#### Centrifugal Device

**Figure 6:**
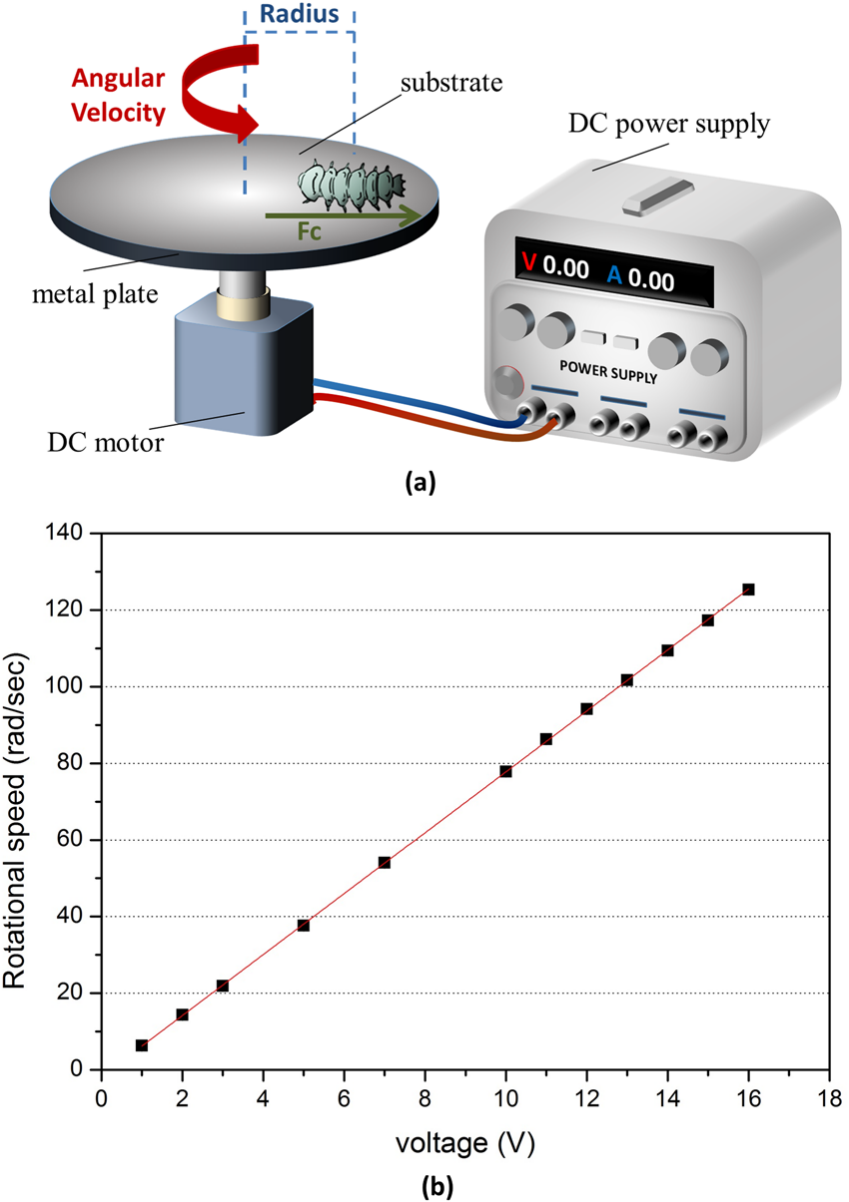
(**a**) Illustration of centrifugal measurement method showing the apparatus of centrifugal measurement is connected to a DC power supply with a metal plate that can be covered by different sand paper, and a DC motor that can rotate the plate. (**b**) The dependence of the rotational velocity of the motor on the applied voltage. The rotational velocity is directly proportional to the applied voltage which can be read on the DC power supply.

In order to evaluate the adhesion capability of aquatic insects, a centrifugal technique was used. According to Gorb *et al*.^25^, this method has been applied to measure the friction and adhesive property for a variety of objects, such as starch microspheres and microcrystalline cellulose^26^, the friction property of skin^27^, the strength of barnacle cement^28^, and insect attachment ability^29^. The main advantage of this measurement is that no pretreatment of insects is necessary, which is suitable for living specimens. The illustration of the centrifugal method is shown in Fig. 6a. A living insect was placed on a metal plate covered with different surfaces (sandpaper, glass slides, Teflon) and then rotated. As the acceleration of rotational velocity increased, the insect would be thrown off at a critical rotational velocity, which was related to the friction force and adhesive force between the attachment devices and the surfaces. The angular speed ω (rad s^−1^) was determined by:

1 $${\rm{\omega }}=\frac{1}{30}\pi \nu $$

where *ν* (rpm) was rotational speed. The centrifugal force *F*_*c*_ was calculated as:

2 $${F}_{c}={mr}{\omega }^{2}$$

where mass *m* (kg) and radius of the position of the insects from the rotor center *r* (m) were measured.

We set up the centrifugal device composed of a metal plate, a DC-motor (024 CXR, with gearhead, Faulhaber), a DC power supply (GDP-23036, 0–30 V, 3 A), as shown in Fig. 6a. The nominal voltage of the DC motor is 24 V. Voltage versus rotational velocity was determined by using a tachometer. As shown in Fig. 6b, voltage and rotational velocity has a linear relationship, which can be used to obtain the corresponding rotational velocities at different values of voltage.

The *Blepharicera* sp. larvae were placed at the position of 4 cm from the center of the circle substrate. The mass of *Blepharicera* sp. larvae was ~25 mg. Adhesion tenacity of the *Blepharicera* sp. larvae was measured on substrates with different surface roughness and wettability. For each surface condition, three individual *Blepharicera* sp. larvae were used and tested for a total of five times.

### Surface Characterization of Substrates

We prepared eight different substrates to evaluate the effect of surface roughness and wettability on the adhesion of sucker. Six of them were sandpaper P80, P120 and P240 (from rougher to smoother) and their corresponding PDMS replicas. The other two were glass slide and Teflon. We used the glass slide and Teflon as a comparison group of substrates because they both were smooth (<5 μm), but had significantly different surface wettabilities. The glass slide was hydrophilic, with contact angle ~30°, while the Teflon was hydrophobic, with contact angle ~105°.

Sandpaper (grade P80, P120, P240, from rougher to smoother surfaces with increasing numbers), glass slides, and Teflon sheet were prepared and served as substrates for centrifugal measurement. The roughness was determined with a α-step surface profiler (Veeco Instruments Inc., Dektak150, Plainview, New York, USA). We also measured the contact angles on the substrate to analyze surface characteristic by using a contact angle meter (Sindatek Instruments Co., Ltd, Model 100SB, Taipei, Taiwan).

In order to evaluate the influence of surface wettability (hydrophilic or hydrophobic) on adhesion with controlled roughness, we synthesized a series of hydrophobic replicated substrates using Polydimethylsiloxane (PDMS) to compare with the hydrophilic sandpaper. PDMS elastomer (Sylgard184 Silicone Elastomer) has two liquid components. When these two liquid components are thoroughly mixed with a 10:1 ratio, the mixture cures to a flexible elastomer. The PDMS mixture was then poured into a container lined with sandpaper. After PDMS hardened from viscous condition, it replicated the surface morphology of sandpaper and obtained similar roughness of the polishing paper while becoming hydrophobic.

The roughness and contact angles of these substrates have been measured, as shown in Supplementary Figure S2. Figure S2i–p shows the contact angles of different grade sandpaper and their replicated PDMS substrates. Contact angles of the slide glass and Teflon sheet were also measured. The contact angles on the sandpaper are smaller (more hydrophilic) than its replicated PDMS substrates (more hydrophobic). For the roughness measurement, P80 is the roughest whereas the glass slide is the smoothest (Fig. S2a–e).

Replicas have similar roughness compared to their original mold sandpaper. From the contact angle measurement, it shows that the PDMS replicas are hydrophobic (>115°) and the sandpaper is more hydrophilic than their replicas (Fig. S2i–p).

## Supplementary information

Supplementary information.

Supplementary information 2.

Supplementary information 3.

Supplementary information 4.

Supplementary information 5.

Supplementary information 6.

## References

[1] Ward, J. V. Aquatic Insect Ecology, Part I, Biology and Habitat. 456 (John Wiley and Sons, 1992).

[2] Ditsche P, Summers AP (2014). Aquatic versus terrestrial attachment: Water makes a difference. Beilstein J. Nanotechnology.

[3] Nachtigall, W. Biological mechanisms of attachment. (Springer-Verlag, 1974).

[4] Hofender H (1927). Über die larven der Blepharoceriden und ihren merkwürdigen Anheftungsapparat. Verh. Zool.-Botan. Ges. Wien.

[5] Rietschel P (1961). Bau, Funktion und Entwicklung der Haftorgane der Blepharoceridenlarven. Z. Morphol. Ökol. Tiere.

[6] Hermann HR, Mullen MA, Wallace IB (1975). Suction discs of *Blepharocera separata*. J. Georgia Entomol. Soc..

[7] Courtney GW (2000). Revision of the net-winged midges of the genus *Blepharicera* macquart (diptera: blephariceridae) of eastern North America. The Entomological Society of Washington.

[8] Lin AYM, Brunner R, Chen PY, Talke FE, Meyers MA (2009). Underwater adhesion of abalone: The role of van der Waals and capillary forces. Acta Materialia.

[9] Kier, W. M. & Smith, A. M. The structure and adhesive mechanism of octopus suckers. *Integrative and Comparative Biology* **42**, 1146–1153 (2002). 10.1093/icb/42.6.1146 21680399

[10] Hou, J., Wright, E., Bonser, R. H. C. & Jeronimidis, G. Development of biomimetic squid-inspired suckers. *Bionic Engineering* **9**, 484–493 (2012).

[11] Yung-Chieh Chuang, Haw-Kai Chang, Guan-Lin Liu & Po-Yu Chen. Climbing upstream: Multi-scale structural characterization and underwater adhesion of the Pulin river loach (Sinogastromyzon puliensis). *Journal of the Mechanical Behavior of Biomedical Materials* **73,** 76-85 (2017). 10.1016/j.jmbbm.2017.01.029 28153482

[12] Kang V, Johnston R, van de Kamp T, Faragó T, Federle W (2019). Morphology of powerful suction organs from blepharicerid larvae living in raging torrents. BMC Zool.

[13] Frutiger, A. Walking on Suckers: New insights into the locomotory behavior of larval net-winged midges (Diptera:Blephariceridae). *Journal of the North American Benthological Society* **17**, 104–120 (1998).

[14] Hynes, H. B. N. The Ecology of Running Waters. (Liverpool University Press, 1970).

[15] Gorb, S. Attachment Devices of Insect Cuticle. (Springer, 2001).

[16] Huber, G. *et al*. Evidence for capillarity contributions to gecko adhesion from single spatula nanomechanical measurements. *PANS*, 16293–16296 (2005). 10.1073/pnas.0506328102 PMC1283435 16260737

[17] Bowden, F. P. & Tabor, D. The Friction and Lubrication of Solids. (Clarendon Press: Oxford, 1986).

[18] P, D.-K., W, B. & JH, K. At which surface roughness do claws cling? Investigations with larvae of the running water mayfly Epeorus assimilis (Heptageniidae, Ephemeroptera). Zoology (Jena) 115, 379-388 (2012). 10.1016/j.zool.2011.11.003 23063107

[19] Beutel R, Gorb SN (2001). Ultrastructure of attachment specializations of hexapods (Arthropoda): evolutionary patterns inferred from a revised ordinal phytogeny. J Zool Sys Evol Res.

[20] Gorb S, Jiao Y, Scherge M (2000). Ultrastructural architecture and mechanical properties of attachment pads in *Tettigonia viridissima* (Orthoptera Tettigoniidae). J. Comp. Physiol. A.

[21] Kier, W. M. & Smith, A. M. The morphology and mechanics of octopus suckers. *Biological Bulletin* **178**, 126–136 (1990). 10.2307/1541971 29314931

[22] Anderson TF (1951). Techniques for the preservation of three dimensional structure in preparing specimens for the electron microscope. Transactions of the New York Academy of Sciences.

[23] Karnovsky, M. J. A formaldehyde-glutaraldehyde fixative of high osmolarity for use electron microscopy. *J. Cell Biol* **27**, 137–138A (1965).

[24] Hayat, M. A. Principles and Techniques of Electron Microscopy, Biological Applications. *Third Edition edn*, (CRC Press, 1989).

[25] Gorb S, Gorb E, Kastner V (2001). Scale effects on the attachment pads and friction forces in syrphid flies (Diptera, Syrphidae). J Exp Bio.

[26] Podczeck F, Newton J (1995). Development of an ultracentrifuge technique to determine the adhesion and friction properties between particles and surfaces. J. Pharmac. Sci..

[27] Highley DR, Coomey M, DenBeste M, Wolfram LJ (1977). Frictional properties of skin. J. Invest. Dermatol..

[28] Dougherty WJ (1990). Barnacle adhesion: reattachment of the adult barnacle *Chthamalus fragilis* Darwin to polystyrene surfaces followed by centrifugational shearing. J. Crust. Biol..

[29] Federle W, Rohrseitz K, Hölldobler B (2000). Attachment forces of ants measured with a centrifuge: better ‘wax-runners’ have a poorer attachment to a smooth surface. J. Exp. Biol..

