# Supplementary material for: Reversible Underwater Adhesion: The Unique C-shaped Suckers of Net-winged Midge Larvae (Blepharicera sp.)
Source: Sci Rep. 2020 Jun 10;10:9395. doi: 10.1038/s41598-020-66268-3 (PMC7286893; doi:10.1038/s41598-020-66268-3)
Supplement: Supplementary file 1 — Supplementary information. [file 41598_2020_66268_MOESM1_ESM.doc]

Supporting Information

Reversible Underwater Adhesion: The Unique C-shaped Suckers of Net-winged Midges (*Blepharicera* sp.)

**Guan-Lin Liu, Haw-Kai Chang, Yung-Chieh Chuang, Yu-Min Lin and Po-Yu Chen***

Department of Materials Science and Engineering, National Tsing Hua University, Hsinchu 101, Sec. 2, Kuang-Fu Rd., Hsinchu 30013, Taiwan

*Corresponding author: poyuchen@mx.nthu.edu.tw

**Figures:**


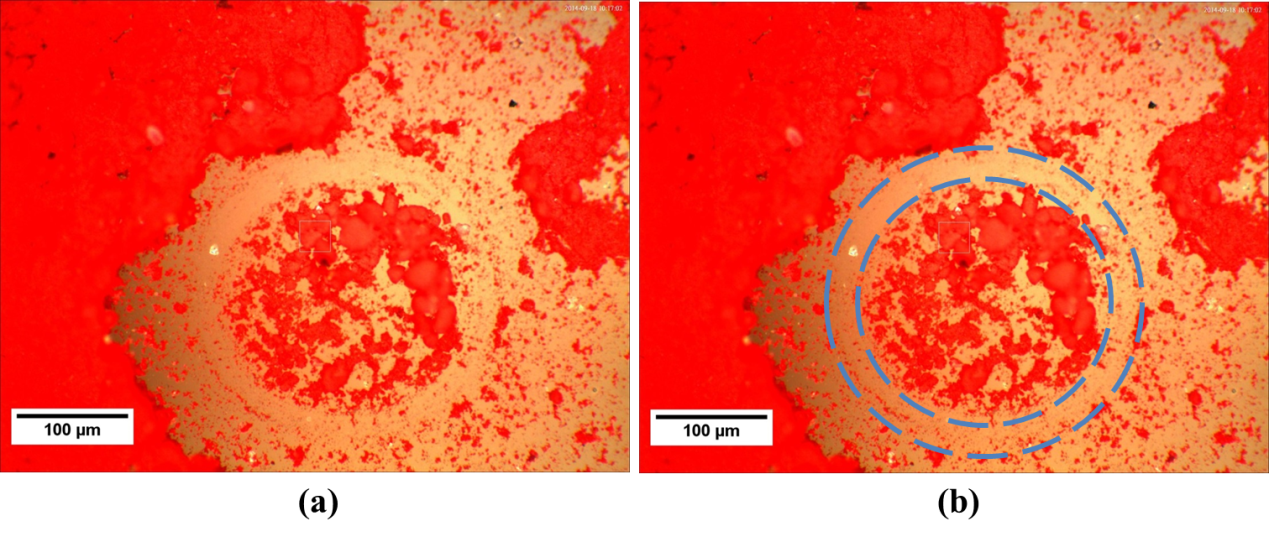


**Figure S1.** The footprint of the larva of *Blepharicera* sp. marked by red ink. (a) Pattern of the larva of *Blepharicera* sp. footprint. (b) Dashed double rings indicate tighter contact between the inward setae and the glass surface.


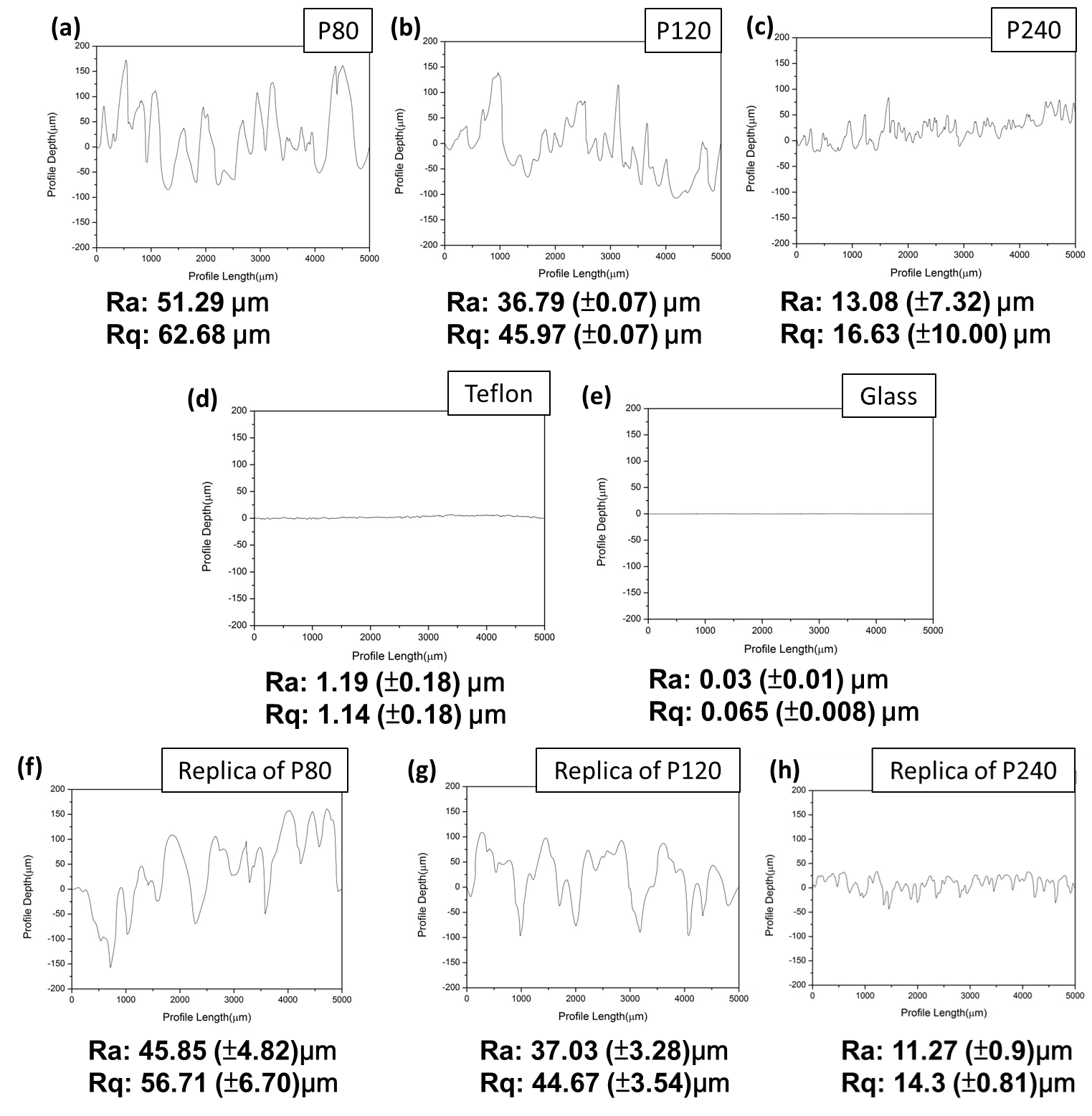


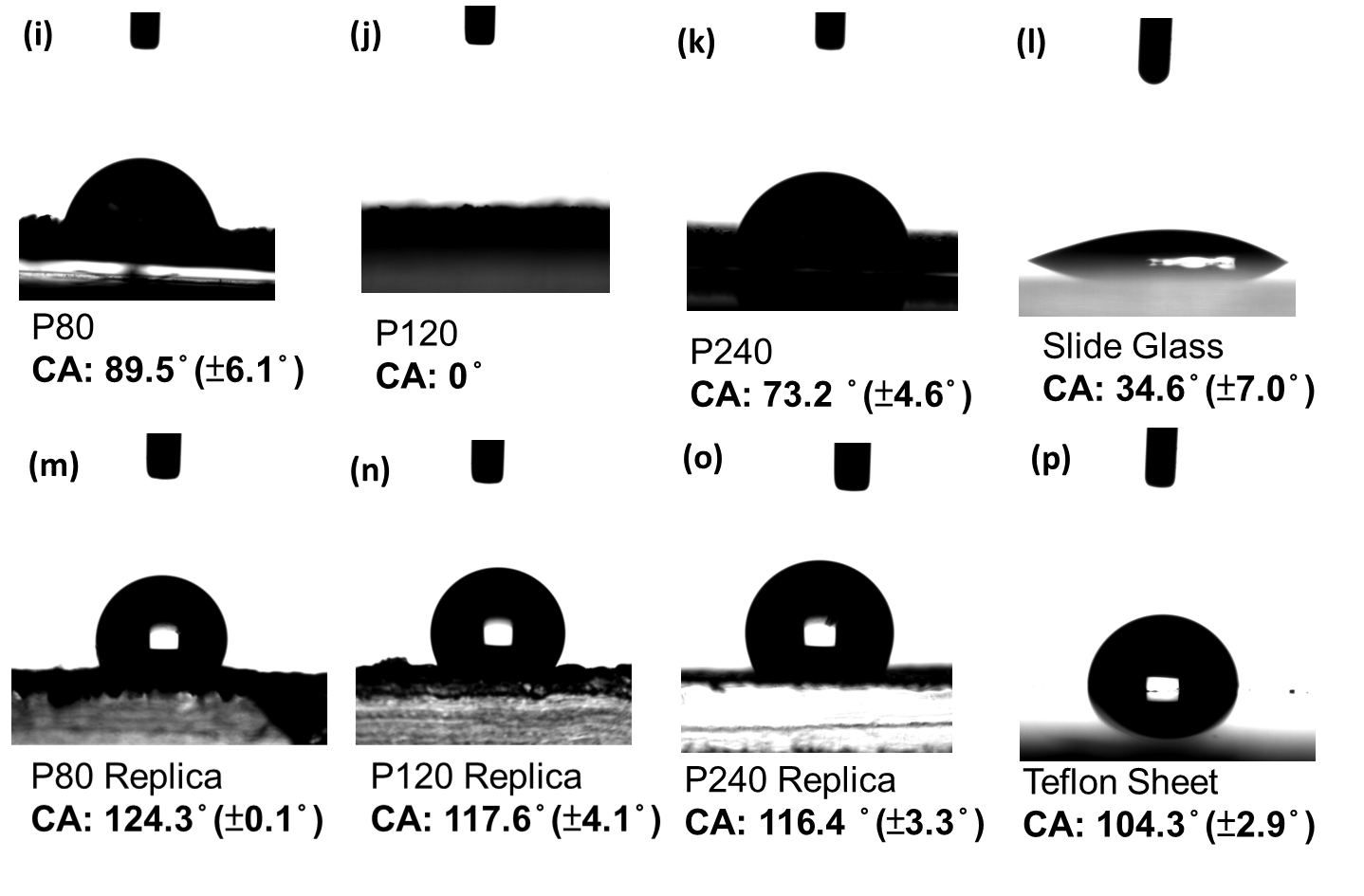


Figure S2. Cross-sectional morphology and roughness of different substrates: (a) P80, (b) P120, (c) P240 sand papers (d) Teflon sheet, (e) glass slide (f) PDMS Replica of P80, (g) PDMS Replica of PDMS P120, (h) PDMS Replica of P240. Contact angle images and measurements of water droplets on (i) P80, (j) P120, (k) P240 sand papers, (l) glass slides, (m) PDMS replica of P80, (n) PDMS replica of P120, (o) PDMS replica of P240, (p) Teflon sheet.

**Videos:**


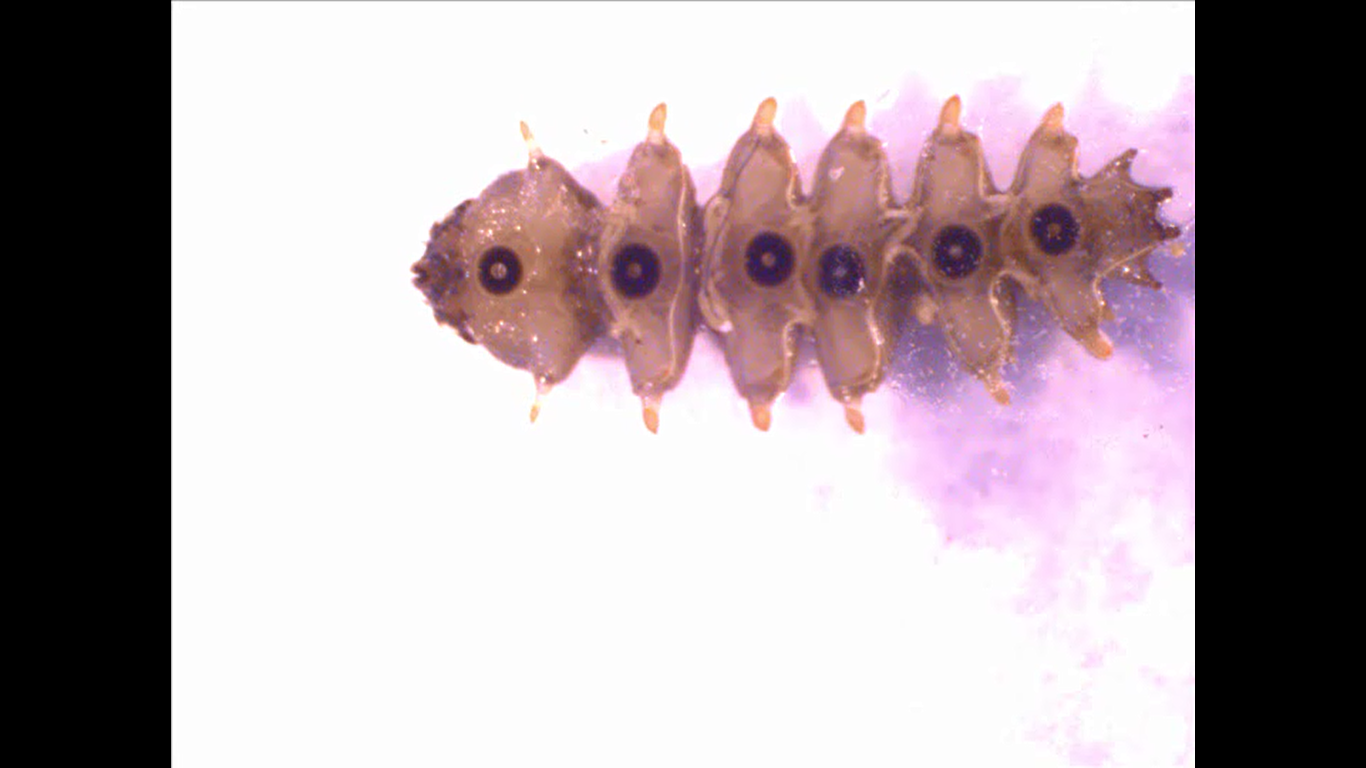


**Video S1.** Forward movement of the larva of *Blepharicera* sp. with one of the suckers detaching and other five suckers attaching to the surface.

**
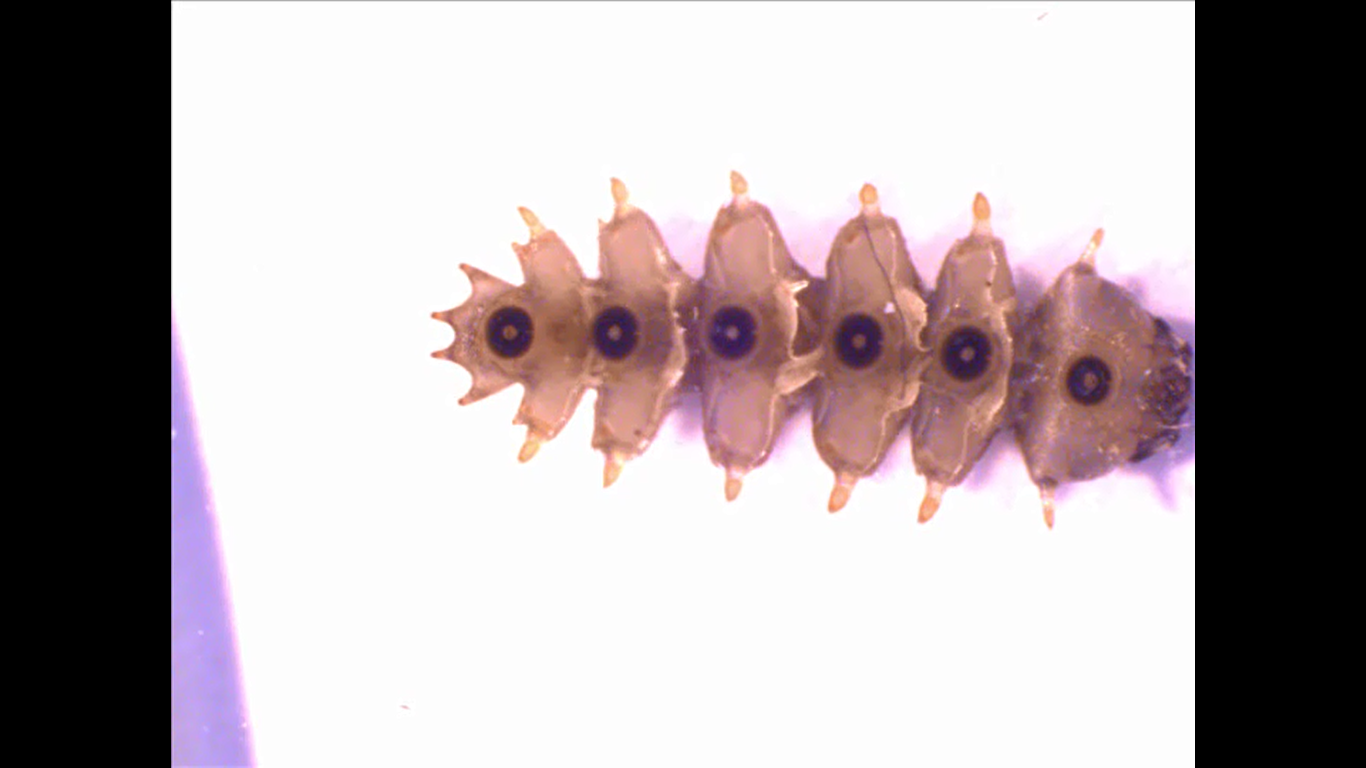
**

**Video S2.** Backward and side motions of the larva of *Blepharicera* sp., are not as fluent and efficient as the forward movement.


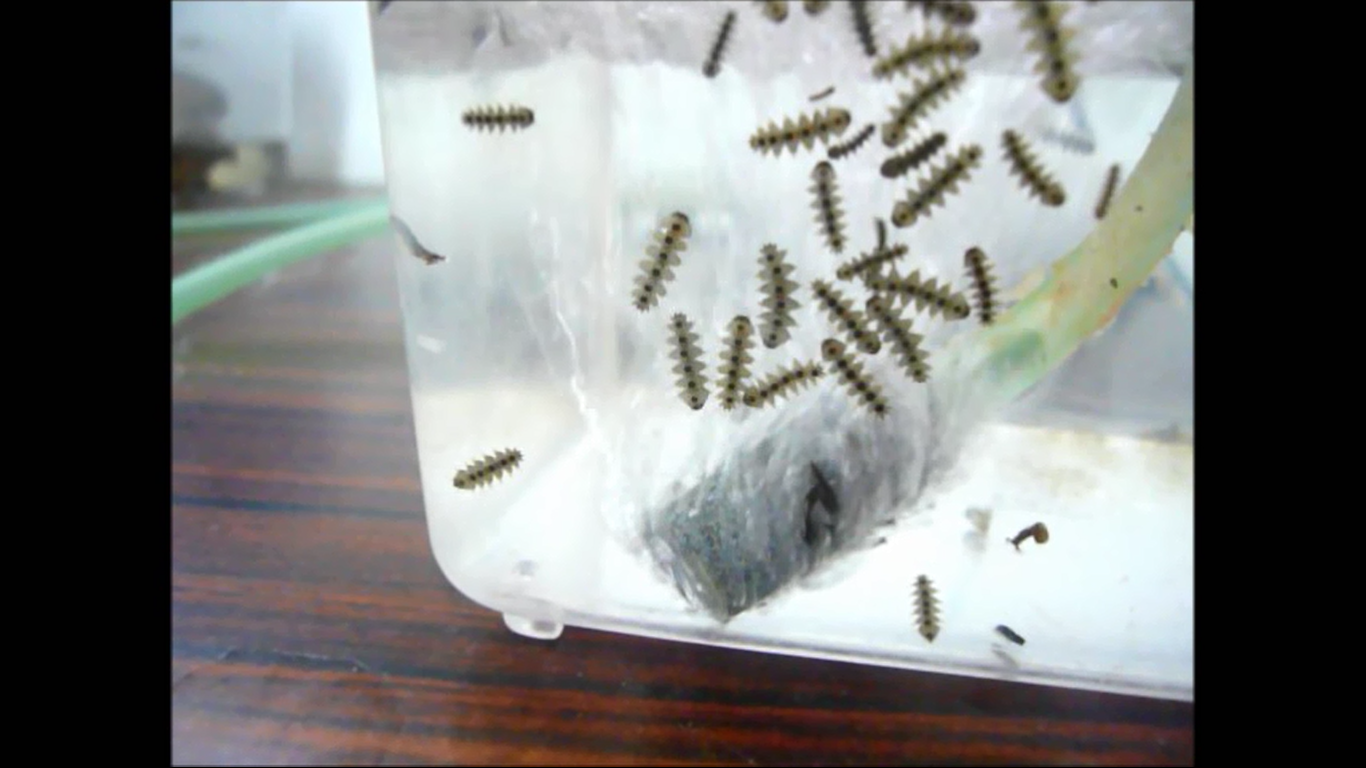


**Video S3.** Rapid side motion by alternatively moving anterior and posterior suckers when the larvae of *Blepharicera* sp. encounter stimulation.


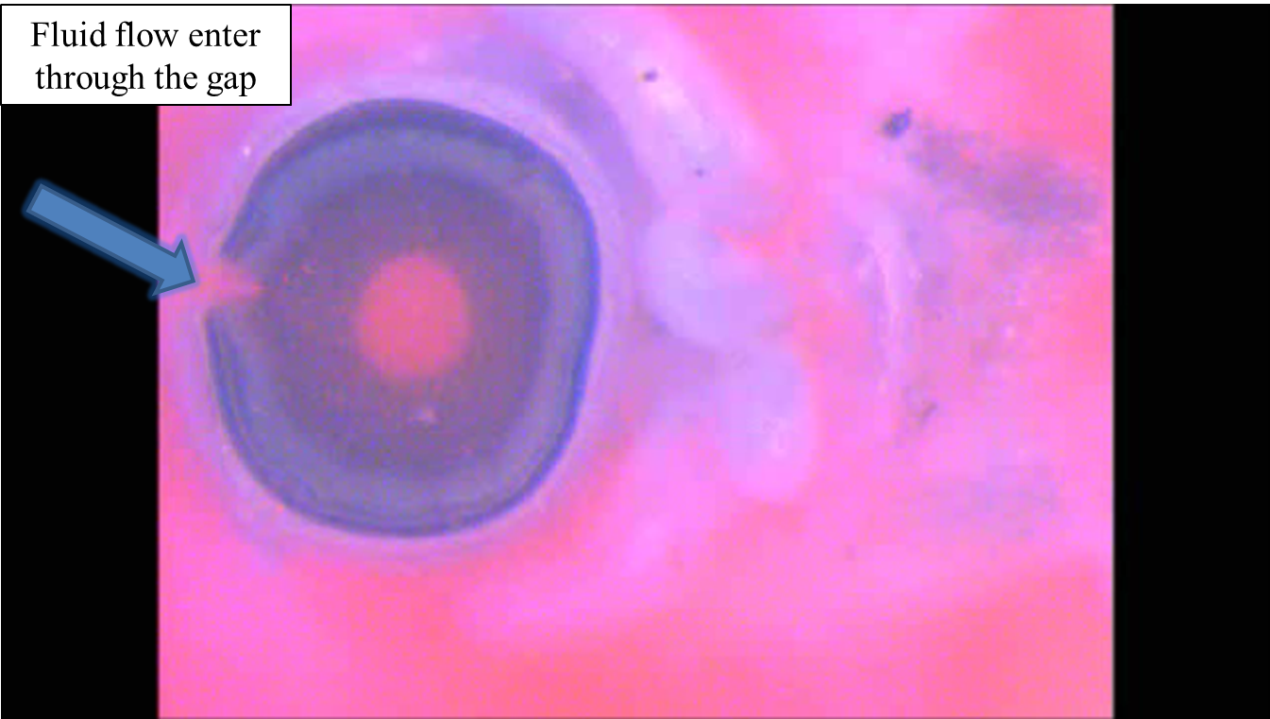


**Video S4.** Video shows fluid entering the sucker through the nick.


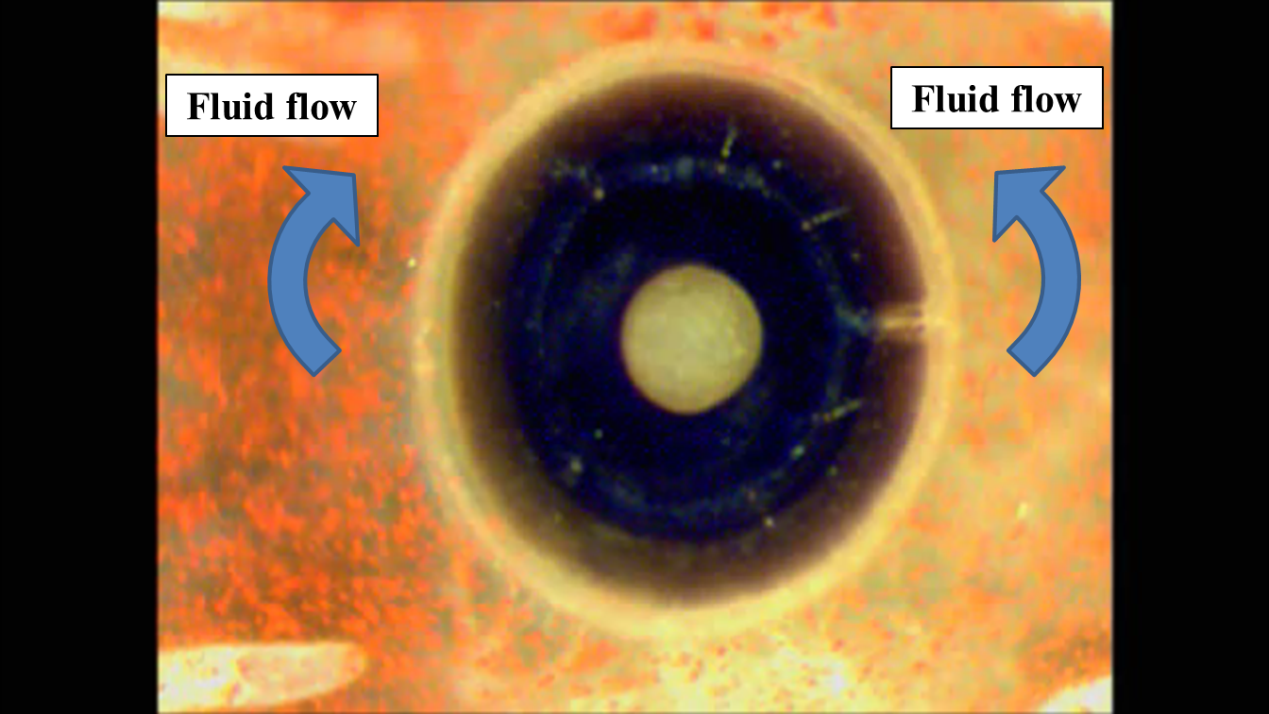


**Video S5.** Video shows fluid flowing along the periphery of the sucker and cannot enter the sucker. The nick is sealed to prevent the invasion of fluid and remain the inner reduced pressure of the sucker.
